# Environmental and Population Studies Concerning Exposure to Pesticides in Iran: A Comprehensive Review

**DOI:** 10.5812/ircmj.13896

**Published:** 2013-12-05

**Authors:** Sara Mostafalou, Somayyeh Karami-Mohajeri, Mohammad Abdollahi

**Affiliations:** 1 Department of Toxicology and Pharmacology, Faculty of Pharmacy and Pharmaceutical Sciences Research Center, Tehran University of Medical Sciences, Tehran, IR Iran; 2 Department of Toxicology and Pharmacology, Faculty of Pharmacy, Kerman University of Medical Sciences, Kerman, IR Iran

**Keywords:** Pesticides, Iran, Environmental, Population

## Abstract

Pesticides are widely used in Iranian agriculture and this has made a major toxicological concern among health professionals. The objective of this study is to explore national data about pesticides toxicity. All relevant databases such as Google Scholar, PubMed, and Scopus in a time period of 1960 to 2012 were searched for the keywords “Pesticides, Iran, Environment, and Population studies”. A total of 57 studies were found relevant and then included into study. Almost all non-experimental studies carried out in Iran were classified into two main categories of residue assessment in different samples and toxic effects on human. Depending on the dose and duration of exposure, toxic effects of pesticides have been studied in two classifications including acute toxicity or acute poisoning and chronic toxicity. High extent of pesticides have been used during the past decade in Iran while no enough proper studies were done to explore their possible toxic effects in the environment and the people.

## 1. Context

**Figure 1.**
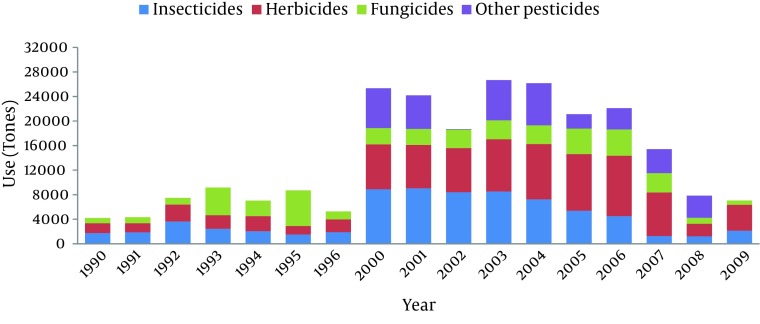
The Rate of Pesticides’ Use in Iran During 1990-2009 Data were extracted from Food and Agriculture Organization of the United Nations (www.fao.org).

Pesticides are considered as a main tool in modern agriculture regarding their potential to control pests and vectors of diseases but knowing their hazards for environment and human health has given rise to controversy on the excessive use of these chemicals. They constitute a large group of chemicals which upon their target are classified into different subgroups like insecticides, herbicides, fungicides, etc. Pesticides dependent agriculture and their widespread use have become an issue of concern in Iran as 18.5 million hectares are cultivated by 3.4 million farmers. Insecticides have become the most used pesticides in Iran (33%), followed by herbicides (30%), fungicides (20 %), acaricides (6.2%), rodenticides (3.8%), nematicides (1.5%) and others (5.5%) (www.pan-uk.org). Beginning in 2000, pesticides’ use has been dramatically increased in Iran and reached to the ultimate in 2003-2004 given by annual usage of 27000 tones throughout the country (Figure 1) (www.faostat.fao.org). 

In order to evaluate the burden of pesticide exposure at public level, different types of population studies have been carried out in the country. In this review, we have gathered all and discussed environmental and population studies related to pesticides exposure in Iran in order to have an overview of ways on which pesticide focused researches should take steps, find the lacks of current information in evaluating the level of pesticides burden, and take clues and approach to direct the perspectives.

## 2. Evidence Acquisition

**Figure 2.**
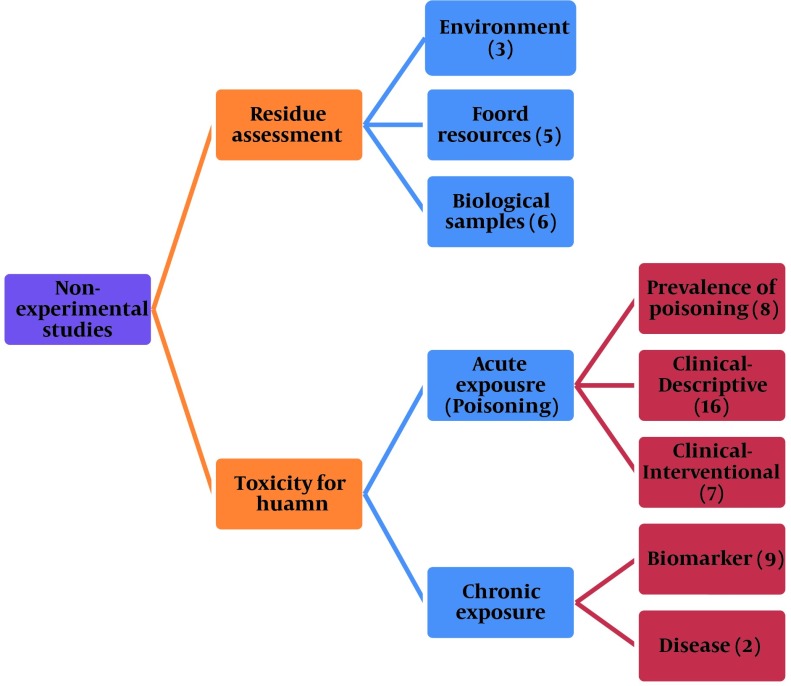
The Main Categories of Pesticides-associated Studies Carried Out in Iran

In order to make a comprehensive review, Google Scholar, PubMed, and Scopus were searched between the years 1960 to 2012 for the keywords “Pesticides, Iran, Environment, and Population studies". A total of 215 articles were found in primary search but after elimination of duplicates or irrelevant papers, only 57 records remained to be reviewed (Table 1). Almost all non-experimental studies carried out in Iran were classified into two main categories of residue assessment in different samples and toxic effects in human. Depending on the dose and duration of exposure, toxic effects of pesticides have been studied in two classifications including acute toxicity or acute poisoning and chronic toxicity. The number of studies in each category is shown in Figure 2. 

**Table 1.** List of Environmental and Population Studies on Health Effect of Pesticides in Iran

Study	Model	Sample	Pesticide	Result
**Sodergren et al. 1978**	Residue assessment	Fish (rivers), Sediments (drainage sys)	DDT, PCBs	High level of DDT, Low level of DDT and PCBs
**Esfahani et al. 2012**	Residue assessment	Sediments and water from wetlands	Chlordane, lindane, endosulfan	Most frequently: chlordane, lindane, and endosulfan
**Fadaei et al. 2012**	Residue assessment	Surface water	Malathion, diazinon	> Allowed limits
**Rezaee et al. 2012**	Residue assessment	Tap and bottled mineral water	Aldicarb, parathion, thiobencarb	< WHO limits
**Shayeghi et al. 2007**	Residue assessment	Drinking water	Malathion, diazinon	> Allowed limit
**Bayat et al. 2011**	Residue assessment	Commercial pasteurized milk	DDT derivatives, PCBs	ADI of PCBs > FAO/WHO limit
**Kalantzi et al. 2001**	Residue assessment	Butter	DDT, DDT isomers, HCB, HCH	DDT, HCB, and HCH > global average
**Jafari et al. 2008**	Residue assessment	Butter	POPs	PCBs, p,p'-DDT and p,p'-DDE > global average 2001
**Dahmardeh et al. 2012**	Residue assessment	Hair samples of pregnant women	DDT, HCB, HCH, and seven PCBs	Significant different on locations and fish consumption
**Hashemy-Tonkabony and Fateminassab 1977**	Residue assessment	Milk of nursing mothers	DDT, BHC, dieldrin	Dieldrin > WHO limit
**Cok et al. 1999**	Residue assessment	Human milk	BHC, HCB, DDT, heptachlor epoxide	BHC, HCB, heptachlor epoxide > FAO/WHO established ADI
**Behrooz et al. 2009**	Residue assessment	Human milk	OCs, PCBs	OC pesticides and PCB > infant daily intake established by Health Canada
**Hashemy-Tonkabony and Soleimani-Amiri 1978**	Residue assessment	Human adipose tissue	OCs	Moderate exposure to HCB, DDT and light exposure to dieldrin
**Burgaz et al. 1995**	Residue assessment	Human adipose tissue	OCs	HCB as the highest contaminant
**Ghazi-Khansari and Oreizi 1995**	Prospective	Poisoning patients	General	Most death due to pesticides (58 %)
**Abdollahi et al. 1997**	Retrospective	Poisoning patients	General	Most death due to pesticides (19.2 %)
**Moghadamnia and Abdollahi 2002**	Epidemiologic	Poisoning patients	General	Most death due to pesticides
**Islambulchilar et al. 2009**	Descriptive, Retrospective	Poisoning patients	General	Most death due to pesticides
**Ahmadi et al. 2010**	Descriptive, Retrospective	Poisoning patients	General	Most death due to pesticides
**Soltaninejad et al. 2007**	Retrospective	Un-survived poisoning	General	Most death due to pesticides; ALP > OP
**Shadnia et al. 2007**	Descriptive, Retrospective	Poisoning patients	General	OPs the main pesticides in poisoning
**Ghazinour et al. 2009**	Retrospective, Descriptive	Poisoning patients	General	Pesticides among the most common causes for parasuicide
**Ranjbar et al. 2005**	Clinical, Descriptive	Poisoning patients	OPs	↓AchE activity, ↑Oxidative stress
**Soltaninejad et al. 2007**	Clinical, Descriptive	Poisoning patients	OPs	Blood ß-glucuronidase as a biomarker at acute poisoning
**Shadnia et al. 2009**	Clinical, Descriptive	Poisoning patients	OPs	Prolonged QTC interval ~ prognosis of poisoning
**Jalali et al. 2010**	Clinical, Descriptive	Poisoning patients	OPs	Distal sensory deficit on EMG
**Noshad et al. 2007**	Clinical, Retrospective	Poisoning patients	OPs	Respiratory failure ~ mortality
**Sabzghabaee et al.**	Clinical, Descriptive	Poisoning patients	Paraquat	PQ dose, vomiting and age as important variables in the mortality
**Soltaninejad et al. 2011**	Clinical, Descriptive, Case report	Poisoning patient	ALP	Met-Hb and Hemolysis as complications
**Shadnia et al. 2010**	Clinical, Descriptive, Case report	Poisoning patients	ALP	Met-Hb and Hemolysis as complications
**Mostafazadeh et al. 2011**	Clinical, Descriptive, Prospective	Poisoning patients	ALP	Met-Hb ~ rate of mortality
**Sanaei-Zadeh 2012**	Clinical, Descriptive	Poisoning patients	ALP	Blood Met-Hb ~ patient outcome
**Mehrpour et al. 2008**	Clinical, Descriptive	Poisoning patients	ALP	Hyperglycemia ~ mortality
**Shadnia et al. 2009**	Clinical, Descriptive	Poisoning patients	ALP	Blood pH ~ patients outcome
**Soltaninejad et al. 2012**	Clinical, Descriptive	Poisoning patients	ALP	Dysrhythmia, elevated ST, prolonged QT, ↑cardiac troponin-T
**Shadnia et al. 2008**	Clinical, Descriptive, Case report	Poisoning patients	ALP	Rapid inhalational absorption, hyperglycemia, surviving
**Shadnia et al. 2011**	Clinical, Descriptive, Prospective	Poisoning patients	ALP	Simplified Acute Physiology Score II to predict outcome
**Saleki et al. 2007**	Clinical, Descriptive	Un-survived poisoning patients	ALP	Cytoplasmic vacuolization of hepatocytes and sinusoidal congestion
**Shadnia et al. 2005**	Clinical, Interventional, Case report	Poisoning patients	ALP	Benefit of coconut oil in the treatment
**Abdollahi et al. 1995**	Clinical Interventional	Poisoning patients	OPs	Use of atropine alone in the treatment of poisoning
**Balali-Mood and Shariat 1998**	Clinical, Interventional	Poisoning patients	OPs	Benefit of PAM + atropine
**Pajoumand et al. 2004**	Clinical, Interventional	Poisoning patients	OPs	Benefit of magnesium sulfate
**Balali-Mood et al. 2005**	Clinical, Interventional	Poisoning patients	OPs	Benefit of sodium bicarbonate
**Shadnia et al. 2011**	Clinical, Interventional	Poisoning patients	OPs	Benefit of NAC
**Afzali and Gholyaf 2008**	Clinical, Interventional	Poisoning patients	Paraquat	Benefit of cyclophosphamide, methylprednisolone
**Tehrani et al. 2012**	Clinical, Interventional	Poisoning patients	ALP	Benefit of NAC
**Abdollahi et al. 1995**	Chronic Biomarkers	Manufacturing workers	OPs	Correlation between plasma AchE and symptoms of poisoning
**Abdollahi et al. 1996**	Cross-sectional	Manufacturing workers	OPs	Correlation between AChE activity in plasma and saliva
**Joshaghani et al. 2007**	Chronic Biomarker	Manufacturing workers	Pesticides	↓ ChE activity in serum and RBC
**Shadnia et al. 2005**	Chronic Biomarker	Manufacturing workers	OPs	↑ Oxidative stress, DNA damage
**Ranjbar et al. 2002**	Chronic Biomarker	Manufacturing workers	OPs	↓ AchE activity, ↑oxidative stress
**Shayeghi et al. 2009**	Chronic Biomarker	Spray workers	OPs, Carbamates	↓ AChE
**Bayrami et al. 2012**	Chronic Biomarker Cross-sectional	Horticulture farmers	OPs	↑ Oxidative stress
**Vandekar et al. 1968**	Chronic Biomarker	Spray workers	O-isopropoxy phenyl methyl carbamate	↓AChE
**Ranjbar et al. 2002**	Chronic Biomarker	Manufacturing workers	Paraquat	↑Oxidative stress
**Zakerinia et al. 2012**	Chronic Disease Retrospective, case control	Exposed people	Pesticides	↑ Risk of non-Hodgkin lymphoma and multiple meyeloma
**Malekirad et al. 2013**	Chronic Diseases, Cross-sectional	Farmers	OPs	↑ Risk of diabetes and neuropsychological disorders

## 3. Reults

### 3.1. Presence of Pesticides Residue

In a study carried out in Mashhad, concentration of aldicarb, parathion, and thiobencarb in the water was measured by gas chromatography and the results were lower than WHO limits (1). Contrary to these results, the amounts of malathion and diazinon were more than allowed limits in the water in Mazandaran (2). There is a same report on the residues of malathion and diazinon in the water collected from places close to agricultural lands in Tehran 1-2 months after spraying (3). The first study on OC pesticides level in water and soil in Iran was performed in 1974. The results implicated high levels of DDT in fish taken from two rivers in southern Iran and low levels of DDT and polychlorinated biphenyls (PCBs) in the samples of sediment from the drainage systems in Tehran (4). Recently, Esfahani et al. (2012) conducted a study from 2009 to 2010 and showed that the most frequent pesticides in sediment and water samples taken from Amir-kalaye wetland were chlordane and then lindane in the summer and lindane and endosulfan in the winter (5).

In a study on commercial pasteurized milk, the levels of DDT derivatives and PCBs were reported to be lesser and higher than the ADI established by FAO/WHO, respectively (6). Furthermore, the concentrations of DDT and its isomers, HCB, and HCH in Iranian butter samples, especially in the central parts of the country were shown to be higher than those found in a global study (7). The highest concentrations of PCB have been found in the samples from Mazandaran province in the North and from industrialized cities such as Tehran, Isfahan and Arak (8). Dahmarde and colleagues measured OC level in hair samples of pregnant women in 2007 and 2008 in Ahvaz and Noushahr cities. Concentration of PCBs was significantly higher in Ahvaz than those in Noushahr and their countryside. Concentration of DDT metabolites was reported to be high in the hair samples taken from countryside of Noushahr (9). Between the years 1974-1976 throughout Tehran area, 131 human milk samples were collected and analyzed for DDT, BHC, and dieldrin by gas-liquid chromatography and the results indicated that the mean concentration of dieldrin exceeded the WHO limit (10). Later, a same study was conducted on 40 human milk samples and daily intake of BHC, HCB, heptachlor epoxide but not DDT for breast-fed children was reported to be higher than ADI established for adults by FAO/WHO Expert Groups (11). In addition, a survey carried out in Nour and Noushahr cities in 2006 indicated that the daily intake of OC pesticides and PCBs for infants via breast milk was above the guideline proposed by Health Canada (12). Only a few reports regarding the levels of OC contaminants in adipose tissue are available for Iran but in a study, HCB was reported as the highest contaminant in human adipose tissues from 1991-1992. In this regard, data collected from a study during 1974-1976 are representative of relatively moderate exposure to DDT and BHC and relatively light exposure to dieldrin (13, 14).

### 3.2. Prevalence of Pesticide Poisoning

In the north of Iran and Tabriz, OP and carbamate insecticides have been reported as the third major agents detected for poisoning and death (15-17). In Tehran, between 1994-1995 and 2003-2004, the result of a couple of studies showed that the most fatal poisoning cases were related to pesticides most commonly aluminum phosphide (ALP) and then OP (18-23).

### 3.3. Biomarkers of Disruption Following Exposure to Pesticides

#### 3.3.1. Organophosphoruses (OPs) and Carbamates

Abdollahi et al. evaluated the level of AChE in samples of healthy and occupationally OPs exposed people in two separate studies and directed well correlation between plasma and erythrocyte ChE (24, 25). A study conducted by Joshaghani and colleagues indicated that the level of ChE activity in the samples of 63 pesticide workers was significantly lower than those of normal group (26). The same results were previously reported by Ranjbar and colleagues in two separate studies on 22 poisoned patients and 45 formulating workers, respectively (27, 28). Measurement of ChE inhibition can be helpful in the management of carbamate and OP poisonings, while in chronic exposures, before/after comparison of this value can produce more acceptable data (29, 30).

Because of variable ChE activity among different persons and lack of pre-exposure data in poisoned patients, the researchers attempt to find other diagnostic factors for management of poisoning. Electrocardiography of OPs poisoned patients was studied and a significant relation between reduction of QT interval and ChE inhibition and mortality rate was found (31). Recently, evaluation of electromyography (EMG) of OPs pesticides poisoning cases with polyneuropathy sings has been conducted in Mashhad by Jalali and colleagues. That study showed a significant dysfunction in sensory rather than motor neurons affecting lower extremities, particularly tibial and personnel nerves, more than upper extremities (32). In another study conducted on four of 98 chemical warfare victims, with severe mustard gas exposure and mean age of 49.8 years, EMG findings revealed positive sharp waves as well as fibrillations. The motor unit action potential defects in these cases indicated presence of axonal polyneuropathy (33).

Genotoxic potential of OPs pesticides was indicated in a study carried out on 21 pesticide formulators. This result was accompanied with a significant elevation in the activity of anterythrocyte antioxidant enzymes such as catalase, superoxide dismutase (SOD) and glutathione peroxidase (34). Increased level of SOD and lipid peroxidation (LPO) was also reported in the samples of 40 horticulture farmers (35) in 22 poisoned patients (28), and in 45 pesticide formulators (27). Blood ß-glucuronidase has been also investigated and suggested as a suitable biomarker for not only chronic but also acute OP poisoning in human (36). In treatment of poisoning, few clinical studies indicated that infusion of high doses of NaHCO3, intravenous magnesium sulfate, and high doses of pralidoxime appears to be beneficial in treatment of patients with OP poisoning (21, 37-39). Although, some reported that atropine alone can be beneficial enough in the management of OP poisoning (40). Furthermore, it has been reported that NAC as a typical antioxidant have beneficial effects on outcome of OP challenged patients when it is used as an adjunct therapy to the standard protocol (41).

#### 3.3.2. Paraquat (PQ)

Mortality rate of PQ poisoning in Iranian hospital was reported 50% (42) of which 81.8% (43) showed a positive correlation with age of patients and also occurrence of vomiting after ingestion (42). Intravenous infusions of cyclophosphamide (15 mg/kg) daily for two days and methylprednisolone 1 g daily for three days decreased the mortality rate of paraquat poisoning by 33.3% (43). In a chronic exposure study on paraquat-formulators, increased LPO and decreased antioxidant power were reported (44).

#### 3.3.3. Aluminum Phosphide (ALP)

There are some reports on the occurrence of mild hyperglycemia following ingestion of ALP (45, 46) and also higher level of blood glucose in non-survivors hypothesizing that there is a correlation between hyperglycemia and the rate of ALP-induced mortality (47-49). Induction of oxidative stress along with protective effect of NAC against LPO markers in the plasma of ALP-poisoned patients have been recently reported (50). Further, ALP has been shown to cause cytoplasmic vacuolization of hepatocytes and sinusoidal congestion in 38 fatal poisoning cases (51). In a retrospective study on 471 ALP-poisoned patients, 93% of cases were self-poisoning. Cardiovascular disorders were the most common signs of poisoning in which correction of blood pH and HCO3 concentration seemed helpful in reduction of mortality (52). Soltaninejad et al. (2012) recorded dysrhythmia, elevated ST segment, and prolonged QT interval in ECG monitoring of 20 patients, as well as a positive serum cardiac troponin-T in 30-40% of patients (53). These researchers developed a simplified acute physiological score according to the demographic data as a prognostic and predictor tool in ALP poisoning (54). An association was found between methemoglobinemia and mortality rate of ALP poisoning which can be treated by methylene blue and ascorbic acid. Beneficial effects of hyperbaric oxygen therapy and exchange blood transfusion have been reported in two ALP poisoned patients who were resistant to methylene blue and ascorbic acid treatments (45, 55-59).

### 3.4. The relationship Between Exposures to Pesticides and Diseases

A retrospective study conducted in Nemazee Hospital, Shiraz in 2007-2008 showed that pesticide-exposed cases were at a higher risk of non-Hodgkin lymphoma and multiple meyeloma (60). Noshad et al. (2007) indicated 12.5% respiratory failures accompanied with 50% mortality in pesticide-poisoned patients admitted to Sina Hospital, Tabriz from 2002 to 2005 (61). A cross-sectional analysis of 187 chronically OP-exposed farmers showed an increased possibility of neuropsychological disorders and metabolic diseases mainly diabetes (62). Another cross-sectional analysis of 40 farmers dealing with pesticides for a long time reported higher incidence of somatization but not psychological disorders (35). Ebrahimi et al. (2007) indicated that almost 50% of total pesticides that used in Fars province of Iran had potential of endocrine disrupting effects such as antiestrogenic, antiandorgenic, antityroidic, antigonadotropin and anti-steroid properties (63, 64). Interfering with function of endocrine system can be the base of many reproductive and developmental disorders as well as sex-related cancers. But there has been no survey exploring the link between exposure to common pesticides in Iran and prevalence of kind of diseases.

## 4. Conclusions

Taken together, all non-experimental studies concerning pesticide exposure in Iran fall into two categories, firstly those dealing with detection of residues in the environment or biological samples and secondly the studies which tried to find useful biomarkers and treatments in people acutely or chronically exposed. For residue assessment in the environment, foods, and biological samples, those pesticides classified as persistent organic pollutants (POPs) like DDT derivatives and the other OCs have been mostly studied, though there are some sporadic reports on the persistence of OPs. However, most of these investigations have shown an alarming point regarding the level of widely used pesticides in the living environment, nutritional resources, and even in the samples taken from people and most seriously from pregnant or nursing women. A main concern regarding pesticides is the high prevalence of their poisoning. In comparison with other agents, pesticides have been classified as the chemicals by which poisoning is the most fatal. One reason is that pesticides are initially designed and synthesized to be toxic for biological systems. In this regard, some therapeutic approaches have been investigated for pesticides poisoning in clinic and valuable results are got.

To evaluate the effects of pesticides on human health, some valuable studies have been carried out on people chronically and occupationally exposed to these chemicals. In this respect, manifestation of the main biomarkers like decreased activity of ChE enzyme and induction of oxidative stress in accessible samples were the main findings. Oxidative stress and accompanying pathways are the main characteristics of disrupted cellular homeostasis and disease process. Moreover the relation between chronic exposure to pesticides and different types of human disease is being more uncovered (65). Except one case-control study concerning higher risk of non-Hodgkin lymphoma and multiple meyeloma in pesticides-exposed people, there is no other investigation on the strength of the link between exposure to pesticides and incidence of diseases in Iran. According to high extent of pesticides use during the past decade and lack of enough evidence regarding the effects of these compounds on the community, Iranian researchers need to pay more attention to diseases which are more probable to progress in the regional people in association with pesticides.

## References

[1] Rezaee R, Hassanzadeh-Khayyat M, Mehri F, Khashyarmanesh Z, Moallemzadeh H, Karimi G (2012). Determination of parathion, aldicarb, and thiobencarb in tap water and bottled mineral water in Mashhad, Iran.. Drug Chem Toxicol..

[2] Fadaei A, Dehghani MH, Nasseri S, Mahvi AH, Rastkari N, Shayeghi M (2012). Organophosphorous pesticides in surface water of Iran.. Bull Environ Contam Toxicol..

[3] Shayeghi M, Khoobdel M, Vatandoost H (2007). Determination of organophosphorus insecticides (malathion and diazinon) residue in the drinking water.. Pak J Biol Sci..

[4] Sodergren A, Djirsarai R, Gharibzadeh M, Moinpour A (1978). Organochlorine residues in aquatic environments in Iran, 1974.. Pestic Monit J..

[5] Esfahani AN, Hassani AH, Farshchi P, Morowati M, Moatar F, Karbassi A (2012). Assessment and investigation on the fate of organochlorine pesticides in water and sediments of international Amir-kalaye wetland in north of Iran.. Bull Environ Contam Toxicol..

[6] Bayat S, Esmaili Sari A, Bahramifar N, Younesi H, Dahmarde Behrooz R (2011). Survey of organochlorine pesticides and polychlorinated biphenyls in commercial pasteurized milk in Iran.. Environ Monit Assess..

[7] Kalantzi OI, Alcock RE, Johnston PA, Santillo D, Stringer RL, Thomas GO (2001). The global distribution of PCBs and organochlorine pesticides in butter.. Environ Sci Technol..

[8] Jafari A, Moeckel C, Jones KC (2008). Spatial biomonitoring of persistent organic pollutants in Iran: a study using locally produced butter.. J Environ Monit..

[9] Dahmardeh Behrooz R, Barghi M, Bahramifar N, Esmaili-Sari A (2012). Organochlorine contaminants in the hair of Iranian pregnant women.. Chemosphere..

[10] Hashemy-Tonkabony SE, Fateminassab F (1977). Chlorinated pesticide residues in milk of Iranian nursing mothers.. J Dairy Sci..

[11] Cok I, Karakaya AE, Afkham BL, Burgaz S (1999). Organochlorine pesticide contaminants in human milk samples collected in Tebriz (Iran).. Bull Environ Contam Toxicol..

[12] Behrooz RD, Sari AE, Bahramifar N, Ghasempouri SM (2009). Organochlorine pesticide and polychlorinated biphenyl residues in human milk from the Southern Coast of Caspian Sea, Iran.. Chemosphere..

[13] Burgaz S, Afkham BL, Karakaya AE (1995). Organochlorine pesticide contaminants in human adipose tissue collected in Tebriz (Iran).. Bull Environ Contam Toxicol..

[14] Hashemy-Tonkabony SE, Soleimani-Amiri MJ (1978). Chlorinated pesticide residues in the body fat of people in Iran.. Environ Res..

[15] Ahmadi A, Pakravan N, Ghazizadeh Z (2010). Pattern of acute food, drug, and chemical poisoning in Sari City, Northern Iran.. Hum Exp Toxicol..

[16] Moghadamnia AA, Abdollahi M (2002). An epidemiological study of poisoning in northern Islamic Republic of Iran.. East Mediterr Health J..

[17] Islambulchilar M, Islambulchilar Z, Kargar-Maher MH (2009). Acute adult poisoning cases admitted to a university hospital in Tabriz, Iran.. Hum Exp Toxicol..

[18] Ghazinour M, Emami H, Richter J, Abdollahi M, Pazhumand A (2009). Age and gender differences in the use of various poisoning methods for deliberate parasuicide cases admitted to loghman hospital in Tehran (2000-2004).. Suicide Life Threat Behav..

[19] Soltaninejad K, Faryadi M, Sardari F (2007). Acute pesticide poisoning related deaths in Tehran during the period 2003-2004.. J Forensic Leg Med..

[20] Shadnia S, Esmaily H, Sasanian G, Pajoumand A, Hassanian-Moghaddam H, Abdollahi M (2007). Pattern of acute poisoning in Tehran-Iran in 2003.. Hum Exp Toxicol..

[21] Rahimi R, Nikfar S, Abdollahi M (2006). Increased morbidity and mortality in acute human organophosphate-poisoned patients treated by oximes: a meta-analysis of clinical trials.. Hum Exp Toxicol..

[22] Abdollahi M, Jalali N, Sabzevari O, Hoseini R, Ghanea T (1997). A retrospective study of poisoning in Tehran.. J Toxicol Clin Toxicol..

[23] Ghazi-Khansari M, Oreizi S (1995). A prospective study of fatal outcomes of poisoning in Tehran.. Vet Hum Toxicol..

[24] Abdollahi M, Balali-Mood M, Akhgari M, Jannat B, Kebriaeezadeh A, Nikfar S (1996). A survey of cholinesterase activity in healthy and organophosphate-exposed populations.. Irn J Med Sci..

[25] Abdollahi M, Jalali N, Jafari A (1995). Organophosphate-induced chronic toxicity in occupationally exposed workers.. Medical Journal of the Islamic Rpublic of Iran..

[26] Joshaghani HR, Ahmadi AR, Mansourian AR (2007). Effects of occupational exposure in pesticide plant on workers' serum and erythrocyte cholinesterase activity.. Int J Occup Med Environ Health..

[27] Ranjbar A, Pasalar P, Abdollahi M (2002). Induction of oxidative stress and acetylcholinesterase inhibition in organophosphorous pesticide manufacturing workers.. Hum Exp Toxicol..

[28] Ranjbar A, Solhi H, Mashayekhi FJ, Susanabdi A, Rezaie A, Abdollahi M (2005). Oxidative stress in acute human poisoning with organophosphorus insecticides; a case control study.. Environ Toxicol Pharmacol..

[29] Shayeghi M, Nasirian H, Nourjah N, Baniardelan M, Shayeghi F, Aboulhassani M (2009). Cholinesterase activity among spray workers in Iran.. Pak J Biol Sci..

[30] Vandekar M, Hedayat S, Plestina R, Ahmady G (1968). A study of the safety of O-isopropoxyphenylmethylcarbamate in an operational field-trial in Iran.. Bull World Health Organ..

[31] Shadnia S, Okazi A, Akhlaghi N, Sasanian G, Abdollahi M (2009). Prognostic value of long QT interval in acute and severe organophosphate poisoning.. J Med Toxicol..

[32] Jalali N, Balali-Mood M, Jalali I, Shakeri MT (2011). Electrophysiological changes in patients with acute organophosphorous pesticide poisoning.. Basic Clin Pharmacol Toxicol..

[33] Holisaz MT, Rayegani SM, Hafezy R, Khedmat H, Motamedi MH (2007). Screening for peripheral neuropathy in chemical warfare victims.. Int J Rehabil Res..

[34] Shadnia S, Azizi E, Hosseini R, Khoei S, Fouladdel S, Pajoumand A (2005). Evaluation of oxidative stress and genotoxicity in organophosphorus insecticide formulators.. Hum Exp Toxicol..

[35] Bayrami M, Hashemi T, Malekirad AA, Ashayeri H, Faraji F, Abdollahi M (2012). Electroencephalogram, cognitive state, psychological disorders, clinical symptom, and oxidative stress in horticulture farmers exposed to organophosphate pesticides.. Toxicol Ind Health..

[36] Soltaninejad K, Shadnia S, Afkhami-Taghipour M, Saljooghi R, Mohammadirad A, Abdollahi M (2007). Blood beta-glucuronidase as a suitable biomarker at acute exposure of severe organophosphorus poisoning in human.. Hum Exp Toxicol..

[37] Balali-Mood M, Ayati MH, Ali-Akbarian H (2005). Effect of high doses of sodium bicarbonate in acute organophosphorous pesticide poisoning.. Clin Toxicol (Phila)..

[38] Pajoumand A, Shadnia S, Rezaie A, Abdi M, Abdollahi M (2004). Benefits of magnesium sulfate in the management of acute human poisoning by organophosphorus insecticides.. Hum Exp Toxicol..

[39] Balali-Mood M, Shariat M (1998). Treatment of organophosphate poisoning. Experience of nerve agents and acute pesticide poisoning on the effects of oximes.. J Physiol Paris..

[40] Abdollahi M, Jafari A, Jalali N, Balali-Mood M, Kebriaeezadeh A, Nikfar S (1995). A new approach to the efficacy of oximes in the management of acute organophosphate poisoning.. Iran J Med Sci..

[41] Shadnia S, Ashrafivand S, Mostafalou S, Abdollahi M (2011). N-acetylcysteine a novel treatment for acute human organophosphate poisoning.. Int J Pharmacol..

[42] Sabzghabaee AM, Eizadi-Mood N, Montazeri K, Yaraghi A, Golabi M (2010). Fatality in paraquat poisoning.. Singapore Med J..

[43] Afzali S, Gholyaf M (2008). The effectiveness of combined treatment with methylprednisolone and cyclophosphamide in oral paraquat poisoning.. Arch Iran Med..

[44] Ranjbar A, Pasalar P, Sedighi A, Abdollahi M (2002). Induction of oxidative stress in paraquat formulating workers.. Toxicol Lett..

[45] Soltaninejad K, Nelson LS, Khodakarim N, Dadvar Z, Shadnia S (2011). Unusual complication of aluminum phosphide poisoning: Development of hemolysis and methemoglobinemia and its successful treatment.. Indian J Crit Care Med..

[46] Mehrpour Omid, Jafarzadeh Mostafa, Abdollahi Mohammad (2012). Sustavni pregled otrovanja aluminijevim fosfidom.. Arhiv za higijenu rada i toksikologiju..

[47] Mehrpour O, Alfred S, Shadnia S, Keyler DE, Soltaninejad K, Chalaki N (2008). Hyperglycemia in acute aluminum phosphide poisoning as a potential prognostic factor.. Hum Exp Toxicol..

[48] Mehrpour O, Aghabiklooei A, Abdollahi M, Singh S (2012). Severe hypoglycemia following acute aluminum phosphide (rice tablet) poisoning; a case report and review of the literature.. Acta Med Iran..

[49] Sanaei-Zadeh H (2012). Response to "blood levels of methemoglobin in patients with aluminum phosphide poisoning and its correlation with patient's outcome".. J Med Toxicol..

[50] Tehrani H, Halvaie Z, Shadnia S, Soltaninejad K, Abdollahi M (2013). Protective effects of N-acetylcysteine on aluminum phosphide-induced oxidative stress in acute human poisoning.. Clin Toxicol (Phila)..

[51] Saleki S, Ardalan FA, Javidan-Nejad A (2007). Liver histopathology of fatal phosphine poisoning.. Forensic Sci Int..

[52] Shadnia S, Sasanian G, Allami P, Hosseini A, Ranjbar A, Amini-Shirazi N (2009). A retrospective 7-years study of aluminum phosphide poisoning in Tehran: opportunities for prevention.. Hum Exp Toxicol..

[53] Soltaninejad K, Beyranvand MR, Momenzadeh SA, Shadnia S (2012). Electrocardiographic findings and cardiac manifestations in acute aluminum phosphide poisoning.. J Forensic Leg Med..

[54] Shadnia S, Mehrpour O, Soltaninejad K (2010). A simplified acute physiology score in the prediction of acute aluminum phosphide poisoning outcome.. Indian J Med Sci..

[55] Shadnia S, Soltaninejad K, Hassanian-Moghadam H, Sadeghi A, Rahimzadeh H, Zamani N (2011). Methemoglobinemia in aluminum phosphide poisoning.. Hum Exp Toxicol..

[56] Mostafazadeh B, Pajoumand A, Farzaneh E, Aghabiklooei A, Rasouli MR (2011). Blood levels of methemoglobin in patients with aluminum phosphide poisoning and its correlation with patient's outcome.. J Med Toxicol..

[57] Mehrpour O, Singh S (2010). Rice tablet poisoning: a major concern in Iranian population.. Hum Exp Toxicol..

[58] Shadnia S, Mehrpour O, Abdollahi M (2008). Unintentional poisoning by phosphine released from aluminum phosphide.. Hum Exp Toxicol..

[59] Shadnia S, Rahimi M, Pajoumand A, Rasouli MH, Abdollahi M (2005). Successful treatment of acute aluminium phosphide poisoning: possible benefit of coconut oil.. Hum Exp Toxicol..

[60] Soltaninejad K, Nelson LS, Khodakarim N, Dadvar Z, Shadnia S (2011). Unusual complication of aluminum phosphide poisoning: Development of hemolysis and methemoglobinemia and its successful treatment.. Indian J Crit Care Med..

[61] Zakerinia M, Namdari M, Amirghofran S (2012). The Relationship between Exposure to Pesticides and the Occurrence of Lymphoid Neoplasm.. Iran Red Crescent Med J..

[62] Noshad H, Ansarin K, Ardalan MR, Ghaffari AR, Safa J, Nezami N (2007). Respiratory failure in organophosphate insecticide poisoning.. Saudi Med J..

[63] Malekirad AA, Faghih M, Mirabdollahi M, Kiani M, Fathi A, Abdollahi M (2013). Neurocognitive, mental health, and glucose disorders in farmers exposed to organophosphorus pesticides.. Arh Hig Rada Toksikol..

[64] Ebrahimi M, Shamabadi N (2007). Endocrine disrupting chemicals in pesticides and herbicide in Fars Province, Iran.. Pak J Biol Sci..

[65] Zekavat SM (1997). The state of the environment in Iran.. J Dev Soc..

[66] Mostafalou S, Abdollahi M (2013). Pesticides and human chronic diseases: evidences, mechanisms, and perspectives.. Toxicol Appl Pharmacol..

